# Phenolic compounds and epigenetic mechanisms regulating gene expression: effects on human health

**DOI:** 10.1007/s13105-025-01105-7

**Published:** 2025-07-07

**Authors:** Silvia Lorente-Cebrián, André G. V. Costa, J. Andrés Castillo-Rivas, Marta Castro, José Miguel Arbonés-Mainar, Saioa Goñi, Sara Remón, Paula Aranaz, Víctor López, Inmaculada Martín-Burriel, Fermín I. Milagro

**Affiliations:** 1 Departamento de Farmacología, Fisiología y Medicina Legal y Forense, Universidad de Zaragoza, Zaragoza, Spain; 2 Adipocyte and Fat Biology Laboratory (AdipoFat), Unidad de Investigación Traslacional, Hospital Universitario Miguel Servet, Zaragoza, Spain; 3 Instituto Agroalimentario de Aragón-IA2 (Universidad de Zaragoza-CITA), 50013 Zaragoza, Spain; 4 Instituto de Investigación Sanitaria Aragón (IIS-Aragón), Zaragoza, Spain; 5 Department of Pharmacy and Nutrition, Centre of Exact, Natural and Health Sciences, Universidade Federal Do Espírito Santo, Alegre, Brazil; 6 Instituto Aragonés de Ciencias de La Salud (IACS), 50009 Zaragoza, Spain; 7 CIBER Fisiopatología Obesidad y Nutrición (CIBERObn), Instituto Salud Carlos III, Madrid, Spain; 8 Área de Bioquímica, Departamento de Ciencias de La Salud, Universidad Pública de Navarra, 31006 Pamplona, Spain; 9 Departamento de Producción Animal y Ciencia de los Alimentos, Universidad de Zaragoza, Zaragoza, Spain; 10 Departamento de Ciencias de La Alimentación y Fisiología, Centro de Investigación en Nutrición, Universidad de Navarra, 31008 Pamplona, Spain; 11 Instituto de Investigación Sanitaria de Navarra (IdiSNA), 31008 Pamplona, Spain; 12 Departamento de Farmacia, Facultad de Ciencias de La Salud, Universidad San Jorge, 50830 Villanueva de Gállego (Zaragoza), Spain; 13 Laboratorio de Genética Bioquímica (LAGENBIO), Facultad de Veterinaria, Universidad de Zaragoza, Zaragoza, Spain; 14 Centro de Investigación Biomédica en Red Enfermedades Neurodegenerativas (CIBERNED), Instituto de Salud Carlos III, 28029 Madrid, Spain; 15 Human Physiology, Department of Pharmacology, Physiology and Legal and Forensic Medicine, Faculty of Health and Sport Science, Universidad de Zaragoza, University Square No. 3, 22002 Huesca, Spain

**Keywords:** Polyphenols, Phenolic compounds, (gene) transcription, Epigenetics, Methylation, Histone modifications, MicroRNAs

## Abstract

Phenolic compounds are a large class of phytochemicals with relevant physiological effects that are naturally found in plant-origin foods and derived products. Beneficial effects associated with polyphenol consumption are related to their ability to prevent and/or counteract disease features: they exert anti-inflammatory, antioxidant and anticancer effects, as well as protective actions against metabolic diseases. Phenolic compounds and their metabolites can modulate cell function by regulating gene expression. These effects are partially mediated through specific changes in epigenetic mechanisms such as DNA methylation, histone modifications and microRNA (miRNA) expression. Some polyphenols affect DNA methylation and are effective in counteracting deleterious actions induced by inflammatory/pro-oxidant factors, both in in vitro and in vivo settings. Specific mechanisms include modulation of methyl-transferases, whose levels are inhibited upon polyphenols treatment. Some polyphenols are histone deacetylase inhibitors, which prevent transcriptional repression and suppress tumor and inflammation genes by affecting selective regulation of miRNA expression. Their mostly recognized actions as anti-inflammatory and antioxidants seem to be partially mediated through regulation of individual miRNAs. Due to these actions, polyphenols and polyphenol-derived metabolites are under study in clinical and interventional trials for their benefits on inflammation and/or metabolic disorders. In conclusion, phenolic compounds might be an interesting approach to contribute to human homeostasis given their capacity to dynamically regulate epigenetic factors at cellular and systemic level. The present review aims to study available evidence regarding regulatory effects of polyphenols on gene expression, specifically mediated through epigenetic mechanisms.

## Introduction

Dietary components of fruits and vegetables regulate cell metabolism and have important consequences in physiological homeostasis at local and systemic level. Phenolic compounds, traditionally known as polyphenols, are plant secondary metabolites found not only in vegetables and fruits but also in fungi and algae [31]. Structurally, they contain one or more aromatic rings with hydroxyl groups, conferring antioxidant properties by neutralizing reactive oxygen species (ROS) [62, 113]. Early studies considered polyphenols as antioxidant agents against lipid peroxidation. However, these compounds have gained attention for their broader health benefits, contributing to oxidative balance, reducing inflammatory processes [62, 129], and influencing cellular signaling [31, 36, 72, 119].

Polyphenols can be classified into simple phenols, phenolic acids, flavonoids, tannins, stilbenes, coumarins and lignans [31, 62, 88]. From over 8,000 phenolic compounds identified, > 4,000 are flavonoids. Due to the significance of the flavonoid class in food sources, phenolic compounds are often divided into two main groups: a) flavonoids (isoflavones, flavones, flavonols, flavanones, flavononols, flavan-3-ols, chalcones, aurones and anthocyanins) and b) non-flavonoids (phenolic alcohols, phenolic acids, stilbenes, coumarins and lignans) [10, 29, 42].

Regarding flavonoids, they can undergo modifications, such as hydroxylation, methylation, and glycosylation, leading to a wide diversity of flavonoid structures [3]. Fruits, particularly those reddish, purple-red, or blue, known as berries, are the most important sources of flavonoids, with anthocyanins the most representative [23, 37]. Other sources include citrus fruits such as lemon, orange, and tangerine, as well as cherries, grapes, plums, pears, apples, and papaya. Flavonoids are also present in vegetables like peppers, broccoli, onions, garlic, tomatoes, coffee, green tea, rock tea, cocoa, grains, and some seeds [3, 111]. More specifically, the flavonols such as quercetin can be found in apples and onions; the flavones (luteolin) in broccoli; flavanones (naringenin), in citrus fruits; flavan-3-ols, such as catechins, in green and black teas and red wine; isoflavonoids, such as isoflavones (also known as phytoestrogens for their modulation of estrogen receptors), in soybeans; and anthocyanins, in red–purple fruits and vegetables and red wine [3, 95].

As for the non-flavonoid compounds, which include the phenolic acids, it is possible to divide them into: a) hydroxybenzoic acids, such as gallic acid, found in grapes; and b) hydroxycinnamic acids, such as caffeic acid, found in coffee [62, 88]. The tannins are divided into hydrolysable tannins, such as gallotannins, and condensed tannins. However, condensed tannins are better known as proanthocyanidins flavonoidic derivatives structures [29]. The stilbene group includes resveratrol, found in grapes. Finally, the lignans contain various bioactive compounds found, for example, in flaxseeds and sesame seeds [3].

In order to study the effects of polyphenols in vivo, we must consider their bioavailability [62, 88]. Several factors can influence their bioactivity, such as food cultivation (sun exposure, soil type, irrigation, maturation stage), food processing (storage, temperature, cooking methods), and interactions with other food matrix components (fibers, lipids, carbohydrates). Nevertheless, other factors related to the individual status and diet are also important: quantitative amount of food, the variety of fruits and vegetables, the physiological state, and intestinal diseases [31, 42]. Additionally, the bioavailability of these compounds also influences solubility, biotransformation of the compounds, interaction with gut microbiota, and the absorption and availability of transporters in the gastrointestinal tract [62, 88].

Moreover, the significant variability of polyphenols, their complex chemical interactions, and various external factors (environmental or individual), influence their bioavailability and physiological and nutritional impact. For example, a small portion of dietary polyphenols can be absorbed in the small intestine (5–10%), where they are metabolized before entering the systemic circulation [10]. However, a significant portion (90–95%), such as high molecular weight polyphenols [25], is metabolized by the microbiota in the large intestine through deglycosylation, dehydroxylation, and demethylation reactions. This process generates absorbable molecules such as phenolic acids and other aromatic compounds [10, 98] as hydroxyphenylacetic, hydroxyphenylpropionic or hydroxyphenylpropionic acids, which are derived from flavonols, flavones/flavanones and flavanols, respectively [75]. An interesting review of the biotransformation of polyphenols by microbiota has been made by Zhang et al., 2022 [131]. Other polyphenols may be smaller but at least need to be released from their matrix, a process carried out by host enzymes but also by others derived from microbes [47, 110]. In the liver, polyphenol metabolites undergo additional biotransformations such as conjugations, which can even increase their biological activity [59] and may return to the intestine via the enterohepatic cycle [10]. In this context, the intestinal microbiota plays an important role in the absorption of polyphenols, which can determine their bioavailability in the systemic circulation and their effects on the gut [36]. Due to these properties, polyphenols have also been proposed as biomarkers of food intake. Conversely, polyphenols also promote the growth of beneficial bacteria such as *Lactobacillus* and *Bifidobacteria* and reduce the presence of pathogens *(Helicobacter pylori, Staphylococcus sp.*) [87, 98]. Beneficial bacteria produce a variety of metabolites such as short-chain fatty acids (SCFAs) that have been involved in epigenetic changes in several chronic diseases such as obesity among others [60].

Despite the fact that polyphenols undergo extensive transformations in their absorption and metabolism, the inter-individual variability is dependent in ADME (administration, delivery, metabolism, excretion) processes. Additionally, the use of non-standardized methods to measure polyphenols, prevent their current application as food biomarkers [21].

As the typical antioxidants, the action of polyphenols in the body occurs by reducing ROS and other free radicals [10, 36, 42, 98], by hydrogen atom transfer, single-electron transfer, sequential electron transfer through proton loss, and chelation of transition metals [129]. Additionally, polyphenols exhibit indirect actions by inducing the transcription factor nuclear factor erythroid 2-related factor 2 (*Nrf2*) or inhibiting the activation of nuclear factor kappa B (NF-κB), which would reduce levels of pro-inflammatory cytokines and adhesion molecules [31, 84, 129].

Polyphenols have attained particular attention not only for their anti-inflammatory and antioxidant properties but also for their ability to counteract the pathophysiological mechanisms related to disease development, being considered as pleiotropic agents. Overall, the benefits associated with polyphenol consumption relate to anti-inflammatory [6], anti-obesogenic [2], cardio-protective [58], anticancer [123], hypo-lipidemic [4] effects, together with the reduction of insulin resistance-related disorders risk [94]. Although most of the beneficial effects of polyphenols have been described in cancer and metabolic syndrome (MS) features, they could also be of interest in other diseases, such as autoimmune disorders or even aging [56].

Notable studies have reported that polyphenols can directly regulate gene expression at cellular and tissue level (reviewed in [16, 54]). In addition to the well-known mechanisms involving radical scavenging properties or direct interaction with physiological targets, phenolic compounds can modulate nuclear receptors, miRNAs, and enzymatic activity, and epigenetic modifications that influence various signaling pathways [19, 72, 119]. However, the intimate mechanisms are still widely unknown. The latest advances in the nutrigenomics field have revealed that polyphenols regulate gene expression through dynamic non-permanent changes in nucleic acids and thus, they could be considered as key epigenetic regulators [96]. Epigenetic regulation involves transitional, non-permanent alterations in gene expression that confer cell plasticity to adapt to environmental cues [32]. Epigenetic mechanisms widely include changes in DNA methylation, histone modifications and expression of non-coding RNAs, such as microRNAs [125]. In this context, the purpose of this review is to analyze the effects of polyphenols on gene expression regulation through epigenetic mechanisms by thoroughly studying the most recent and currently available scientific evidence in humans and some scientifically related in vitro and in vivo studies.

The methodological approach for the present review was performed in PubMed database and consisted of strategical combination of main keywords (f. i., “polyphenols AND gene transcription”; “polyphenols AND epigenetics”) for global search and restricted to the latest 10 years including in vitro (mainly human-origin cell models), in vivo (animal studies) and clinical (human) trials. Additional and complementary searches were also performed using “polyphenols” and specific epigenetic factors (as secondary keywords). These specific searches were restricted to the latest 5 years (2020–2024): a) “polyphenols and DNA methylation”, b) “polyphenols and histone modifications” which included histones “acetylation” and “phosphorylation” and c) “Polyphenols and microRNAs”, that yield most abundant publications number (235 hits). Finally, the most relevant articles were selected and preferentially considered those carried out in humans and addressing single polyphenols effects in original (non-review) articles. Top relevant basic studies addressing epigenetic function mechanisms were also considered and were not restricted to any particular timeframe.

### Polyphenolic compounds decrease global DNA methylation by inhibiting DNA methyl-transferases (DNMTs)

DNA methylation, a key epigenetic modification, regulates gene expression by adding methyl groups to cytosine residues, often leading to transcriptional repressing [80]. Several polyphenols, have been shown to modulate DNA methylation, thereby reversing deleterious methylation patterns associated with certain diseases such as cancer, metabolic diseases (obesity) and inflammation-related disorders, including neurological diseases [78, 82, 90]. The effects of specific polyphenols on DNA methylation have been addressed in different in vitro and in vivo studies, even involving nutritional interventions in humans. Importantly, most recognized biological actions of polyphenols (antioxidant and anti-inflammatory capacity) seem to be partially mediated through cell-specific mechanisms involving changes in DNA methylation [89]. In most cases, these mechanisms affect DNA methyl-transferases (DNMTs) activity leading to decreased DNA methylation usually with subsequent up/downregulation of functional genes, depending on its physiological role. The regulation of polyphenols on DNMTs could be either mediated through direct (binding) interaction (f. i., epigallocatechin-3-gallate [117] but also as an indirect effects of polyphenols on other signaling pathways such as cellular redox state and/or inflammation (f. i., curcumin [69, 102]). Interestingly, in the absence of deleterious effects, the regulatory actions of bioactive (poly)phenolic compounds on DNMTs remain mostly silenced. Globally, reported studies have shown decreased DNA methylation due to DNMT inhibition upon polyphenols treatment. Single polyphenol molecules that had been linked to biological actions and had been well-reported include resveratrol, ptero-stilbene(s) and curcumin. The most relevant studies and involved mechanisms are detailed in Table 1. However, the magnitude of reported effects in these studies varies greatly. Resveratrol, one of the most well-studied bioactive compounds, decreases DNA methylation specifically inhibiting DNMT expression, which has been observed in different cell types and disease models, including cancer, obesity and non-alcoholic fatty liver disease (NAFLD) [48, 99, 116]. Interestingly, in metabolic disease context (obesity and NAFLD), resveratrol treatment increased lipid turnover by inhibiting lipid accumulation and promoting white adipose tissue (WAT) browning both in in vitro and in in vivo studies, as depicted in Table 1 [48, 99]. These effects translated into specific changes on gene expression, for instance, decreasing fatty acid synthase (*Fas)* and sterol-regulatory element binding protein 1c (*Srebp1c*) [48] and increasing PR domain containing 16 (*Prdm16*) expression [99] at cellular (3T3-L1, HepG2) and tissue level (liver and WAT, respectively). Curcumin also improved liver fibrosis and obesity by decreasing methionine adenosyl-transferase 2 A (MAT2A) expression, a regulatory effect mediated via MAPK pathway (JNK and p38 signaling) which in overall, decreases DNA methylation in isolated hepatic stellate cells and mice [50, 70]. Ptero-stilbene has shown contradictory effects: while Beetch et al*.*, [15] found an increase in DNMT3B activity, thereby increasing DNA methylation, others found that ptero-stilbene reversed DNA methyl-transferase and histone deacetylase expression by specifically decreasing Histone Deacetylases (HDACs) *HDAC1*, *HDAC3* and *HDAC4* expression in human endothelial cells in a hyperglycemic environment [45] (see Table 1). However, these apparent differences might depend on polyphenol type, bioavailability, concentration/dose used, as well as on the experimental design (single molecule *vs*. enriched extracts) and/or clinical context (cancer, metabolism-related or inflammation). Interestingly, polyphenols might also be loaded into specialized nanoparticles to overcome their low bioavailability and absorption [93], resulting in decreased expression of DNMTs and improved antimicrobial and cytotoxic effects. Further studies are depicted in Table 1, being of particular interest those dealing with specific effects of single molecules on DNA methylation levels, as well as those with no effect after polyphenol treatment. Moreover, the differential magnitude of effects found in in vivo studies might rely as well on polyphenols pharmacokinetics and dynamics in different mice strains, disease models, and assessed human conditions. The most relevant outcomes of the analyzed studies are depicted in Table 1.

**Table 1:** Effects of polyphenols on DNA methylation and their influence in several models of human diseases

Polyphenol type/source	Dose or concentration	Epigenetic outcome	Mechanism involved (target gene and/or functional pathway)	Model	Disease	Reference
Apple polyphenols	8-wk (700 mg/kg body weight)	↑ DNA methylation	Adipose hypertrophy	DIO mice	Obesity	[17]
*Aronia melanocarpa* juice (polyphenol-rich)	Daily consumption for 4-wk (100 mL of juice contained 1,177.11 mg of gallic acid, highly represented proanthocyanidins)	↓ DNA methylation	↓LINE1 (a surrogate marker of global DNA methylation)	Human peripheral blood leukocytes	MS features (*n* = 54 subjects with moderate CVD risk). NCT02800967	[103]
Blackberry extract	IC_20_ & IC_50_ for 72 h	↓ DNA methylation (promoter)	Differential effects on DNMTs and HDACs dependent on doses and cell lines: ↓DNMTs (*Dnmt1* and *Dnmt3b*) ↑DNMT (*Dnmt3a)* ↑SIRT1 expression (in HCT116, HT29/219, ↓Cellular differentiation)	Human cancer cells (HCT116, HT29/219, SW480, SW742, and LS180)	Cancer	[106]
Caffeic acid phenethyl esters (CAPE), daidzein, isorhamnetin and genistein	In vitro: 2–20 $\mu$M (IC_50_ < 4.3–7.6 $\mu$M) In vivo: 0.015% CAPE-supplemented diet	↓DNA methylation in vitro and in vivo	In vitro: ↓DNMT1 activity In vivo: Offspring protection	Human cancer (HT29) cells Agouti mice	Human colorectal adenocarcinoma. Maternal exposition	[115]
*Camellia sinensis* extract (catechins 80%) *Morella rubra* extract (myricetin 80%) Flavonoid extract (with added resveratrol) Coffee arabica extract	2-wk treatment: 4 mg/day/animal green tea 2,5 mg/day/animal bayberry 30 mg/day/animal a flavonoid extract (added 4 g/100 mL) 30 mg/day/animal of 150 ml	↑ activity of DNA methyltransferase enzymes Did not prevent hypomethylation in the kidney Flavonoid extract ↑methylation in the liver	↑LINE-1 methylation ↑Glutathione levels ↓Free radical damage Antioxidant, anti-inflammatory properties	Liver, spleen, kidney in mice	N/A (strain used in basic research)	[105]
Epigallocatechin-3-gallate (EGCG)	5 $\mu$M for 24–72 h	Restoration of aberrant expression of epigenetic proteins	↓DNMT1, DNMT3A, DNMT3B, MBD4, HAT1, HMT1, HDAC1 gene and protein expression ↓H3K27me2, H3K27ac levels ↓H3K27me2 and H3K9ac levels	Human kidney epithelial cells (Caki-1, HK-2)	Renal fibrosis (chronic kidney disease)	[52]
Epigallocatechin-3-gallate (EGCG)	5–50 $\mu$M (IC_50_ < 1 $\mu$M) for 12–144 h	↓DNA methylation Reversion of methylation-induced gene silencing	↓DNMT activity (direct binding) ↓CARM1	Human esophageal (KYSE 510), prostate cancer (PC3), colon cancer (H29) cell lines	Cancer	[35, 57]
Curcumin	N/A	↓ DNA methylation	↓MAT2A expression (↓JNK signaling)	Hepatic stellate cell (HSC) from *ob/ob* mice	Genetic obesity and liver fibrogenesis	[70]
Curcumin	4-wk treatment (400 mg/kg body weight, once daily by oral gavage)	Remodeling of DNA methylation, (through methionine adenosyl transferase II, MATII)	↓MAT2B expression (regulatory subunit) via ↓p38 MAPK activation pathway ↓collagen level	Hepatic stellate cell (HSC) and liver fibrosis	Liver fibrosis	[50]
Genistein	2-wks of 250 mg/kg diet of genistein (comparable levels with humans consuming high-soy diets)	↑ DNA methylation	Offspring protection	Maternal supplementation during gestation in mice	Obesity	[28]
Hydroxytyrosol (HT) from extra virgin olive oil	10 $\mu$M HT for 7-days	↑Global DNA methylation	↓endothelin receptor type A gene (EDNRA) expression	Human colorectal cells (Caco-2)	Cancer	[97]
Isoflavones (from soy)	Equivalent to 180 mg/person/day of soy isoflavones (mimics high soy supplementation diet)	↑ DNA methylation	HOXA5, HOXA11, HOXB1 and ABCG5	Blood, liver, fat and muscle biopsies in monkeys	N/A	[49]
Pterostilbene	7 $\mu$M for 4 days	↑DNA methylation	↑DNMT3B activity: ↓PRKCA, TNNT2, DANT2 (↓OCT1 binding)	Breast cancer cells	Cancer	[15]
Pterostilbene	0–100 $\mu$M for 24–48 h	Reversed changes on DNA methyltransferases and histones deacetylases expression. Counteraction of CpG islands hypermethylation (Nrf2 promoter)	↓HDAC1, HDAC3, HDAC4 expression ↑DNMTs expression (1/3A,3B) Methyl-sensitive restriction enzyme effects	Human endothelial cells	Hyperglycemic microenvironment (metabolic diseases)	[45]
Quercetin (Q) with green tea extract (GTE)	1 g of GTE (830 mg of GT polyphenols) with 800 mg of Q (GT + Q) or placebo (GT + PL) for 4-wk	No effect on methylation activity	N/A. No liver toxicity	Prostate tissue or RBCs	Men with prostate cancer (*n* = 31)	[46]
Resveratrol	50 mg/kg/day by gavage for 21 days	↓DNA methylation	↓DNMTs expression (MeCP2, PP1), cleaved caspase 3 ↑SIRT1, BDNF	Cancer (PC12) cells and hippocampus of rats	Cancer	[116]
Resveratrol	2 mg/kg of body weight for 21 days	↓DNA methylation at target sites regions (promoter, intron and exons regions)	↑Slc27a1 and Prdm16 mRNA expression (↑WAT browning)	Inguinal (sc) WAT of neonatal mice and 3T3-L1 adipocytes	Obesity (HFD)	[99]
Resveratrol	In vitro: 10–200 $\mu$M In vivo: HFD (56% kcal of fat) + 0.4% supplementation	↓HFD-induced DNA methylation	↑Nrf promoter activity: ↓TG, ↓FAS, SREBP-1c expression (↓lipid accumulation)	HepG2 and liver of mice	NFALD	[48]
Tannin extract (TE) from maritime pine: Catechin/epicatechin, epigallocatechin, epicatechin gallate and lanostane terpenoids	12.5–500 $\mu$g/mL	↓ Methylation levels	↑caspase 3 activation, ↓Bcl-2 ↓pro-oncogenic proteins UHRF1 and DNMT1	Human cancer cells (HeLa, U2OS cells) & fibroblasts	Cancer	[9]
Tannic acid	IC50 = 500 ug/mL	↓ DNA methylation	↓DNMT1, DNMT3A, DNMT3B expression = 5-methylcytosine levels	HepG2	Liver cancer cells	[93]

*DNMTs*, DNA methyltransferases; *HDACs*, histone deacetylases; *MS*, metabolic syndrome; *RBCs*, red blood cells; *SIRT1*, sirtuin 1

*N/A*, non-applicable or non-available

A key question is whether polyphenols might induce pernicious effects due to their changes in DNA methylation pattern. Most authors have shown polyphenol-induced inhibition of DNMT to be protective against disease development and do not specifically address negative effects of polyphenols on DNA methylation (reviewed in [127]). However, some authors have suggested the induction of tumors due to severe DNMT inhibition and the presence of hypo-methylated DNA [30, 38], which warrants further studies to fully clarify these potentially negative findings, particularly with high doses of polyphenols.

### Polyphenols promote histone acetylation, leading to gene expression activation

Histone modifications play a crucial role in chromatin remodeling and gene expression regulation [13]. Examples of histone modifications include histone acetylation, methylation and phosphorylation. Histone architecture is linked to genome-wide DNA methylation distribution suggesting a tight link between these epigenetic marks, which appear frequently altered in human diseases [64]. Importantly, histone-modifying enzymes, such as histone deacetylases (HDACs), histone acetyltransferases (HAT) or histone methyl-transferases (HMT), constitute therapeutic targets physiologically regulated by bioactive compounds of synthetic or natural origin such as polyphenols. Curcumin, ellagic acid and epigallocathechin-3-gallate (EGCG) have gained considerable attention since they act as potent HDAC inhibitors [16]. HDAC inhibitors prevent the removal of acetyl groups from lysine residues in histones, resulting in increased gene transcription. The effects of polyphenols as HDACs inhibitors have been tested in different cell and animal models with varying conditions depending on polyphenol type, dosing and length treatment (see Table 2). However, recently available studies suggest conflicting results regarding the role of specific polyphenols compounds on histone modifications. While the most relevant results of single-polyphenolic molecules on DNA methylation had been described above, changes on chromatin remodeling, due to histone acetylation, are less studied so far. For instance, specific polyphenol-derived EGCG can directly bind to HDAC enzymes preventing histone H3 and H4 de-acetylation, and the transcriptional repression of target genes. On the other hand, curcumin has been shown to induce the enrichment of H3K27ac mark in the promoter of superoxide dismutase 2 (*Sod2*), which is related to the upregulation of *Sod2* and to an improved phenotype in a neuro-inflammation animal model [124]. Other metabolites derived from polyphenols, such as 3-OH-phenylacetate or 3-OH-phenyl-propionate, inhibit HDAC activity in in vitro models [16] by extending acetylated chromatin status. For example, urolithin C, a gut bacteria-derived product from ellagic acid, decreases TNFα-induced inflammation by inhibiting HAT in monocytes [39]. More details on these effects and mechanisms of single polyphenolic molecules, as well as some well-characterized extracts, are shown in Table 2. Notably, not all studies have reported positive effects of polyphenols or their derived metabolites on improving disease conditions. For instance, Kadosh et al*.*, [53] reported that gallic acid, a polyphenol-derived metabolite produced by gut microbiome, could reinstate transcription factor 4 (TCF4)-chromatin interaction and hyper-activation of Wnt signaling, conferring a malignant phenotype in an in vivo cancer model. In this study, polyphenol-derived metabolites (ellagic acid, urolithin B, gallic acid) induced organoid remodeling stimulating proliferative capacity. This suggests that inhibitors/antagonists of gallic synthesis would be desirable to maintain the tumor suppressive properties of cells (see Table 2). Further investigations are needed to clarify the role of these molecules in cancer and disease progression. Some polyphenols and other polyphenol-derived metabolites acting as HDAC inhibitors have been tested in clinical trials as anti-neoplastic compounds, against neurological disorders or metabolic diseases (reviewed in [43, 73, 104]). Since some HDAC inhibitors have even received approval for their biological use, polyphenols and their derived metabolites hold potential as nutraceutical compounds in future therapies.

**Table 2:** Main actions of polyphenols on regulating histone modifications and their impact in several models of human diseases

Polyphenol type/source	Dose or concentration	Epigenetic outcome	Mechanism involved (target gene and/or functional pathway)	Model	Disease	Reference
Curcumin	1.5–12.5 μM for 72 h	Chromatin remodeling: ↓Histone acetylation	↓HAT activity ↑HDAC expression	Human monocytic cells (THP-1)	Inflammation and diabetic-linked complications	[128]
Curcumin	16-wk of curcumin (400 mg/kg) oral treatment	↑Histone acetylation	↑H3K27 acetylation level (at *Sod2* gene promoter) → ↑*Sod*2 expression	Striatal neurons in rats	Neurological disease	[124]
Ellagic acid (EA)	10 μM EA during adipocyte differentiation (12–14 d)	Chromatin remodeling: ↓Histone acetylation	↑HDAC9 expression → ↓CARM1 (inhibits human adipogenesis)	Human adipocytes (hASCs)	Obesity	[55]
Epigallocatechin-3-gallate (EGCG)	5–20 μM for 72 h or 144 h	↑Histone (H3 and H4) acetylation	↓DNMTs activity (↓DNMT protein expression: DNMT1, DNMT3a, DNMT3b)	Human skin cancer (A431)	Cancer	[81]
Epigallocatechin-3-gallate (EGCG)	5–200 μM for 24 h or 72 h	Chromatin architecture (promotes chromatin relaxation)	↑Histone acetylation (H3K9/14ac, H3ac) ↓HDAC expression	Human endothelial cells (HMEC-1 and HUVEC)	Non-tumor cells, but related to tumor growth (neoangiogenesis and metastasis)	[20]
Muscadine grape phytochemical powder (MGP) containing ellagic acid (EA)	15-wk HFD (60% kcal from fat) + 0.4% MGP (18 mg EA/day)	Chromatin remodeling: ↓Histone acetylation	↑HDAC9 expression → Negative regulation of adipogenesis and lipid accumulation	Primary human adipocytes and human hepatoma cells (Huh7)	Obesity/lipid accumulation	[85]

*CARM1*, coactivator associated arginine methyltransferase 1; *DNMTs*, DNA methyltransferases; *HDACs*, histone deacetylases; *hASC*, human adipose stem cells; *HUVEC*, human umbilical vein endothelial cells; *Sod2*, superoxide dismutase 2

*N/A*, non-applicable or non-available

**Fig. 1:**
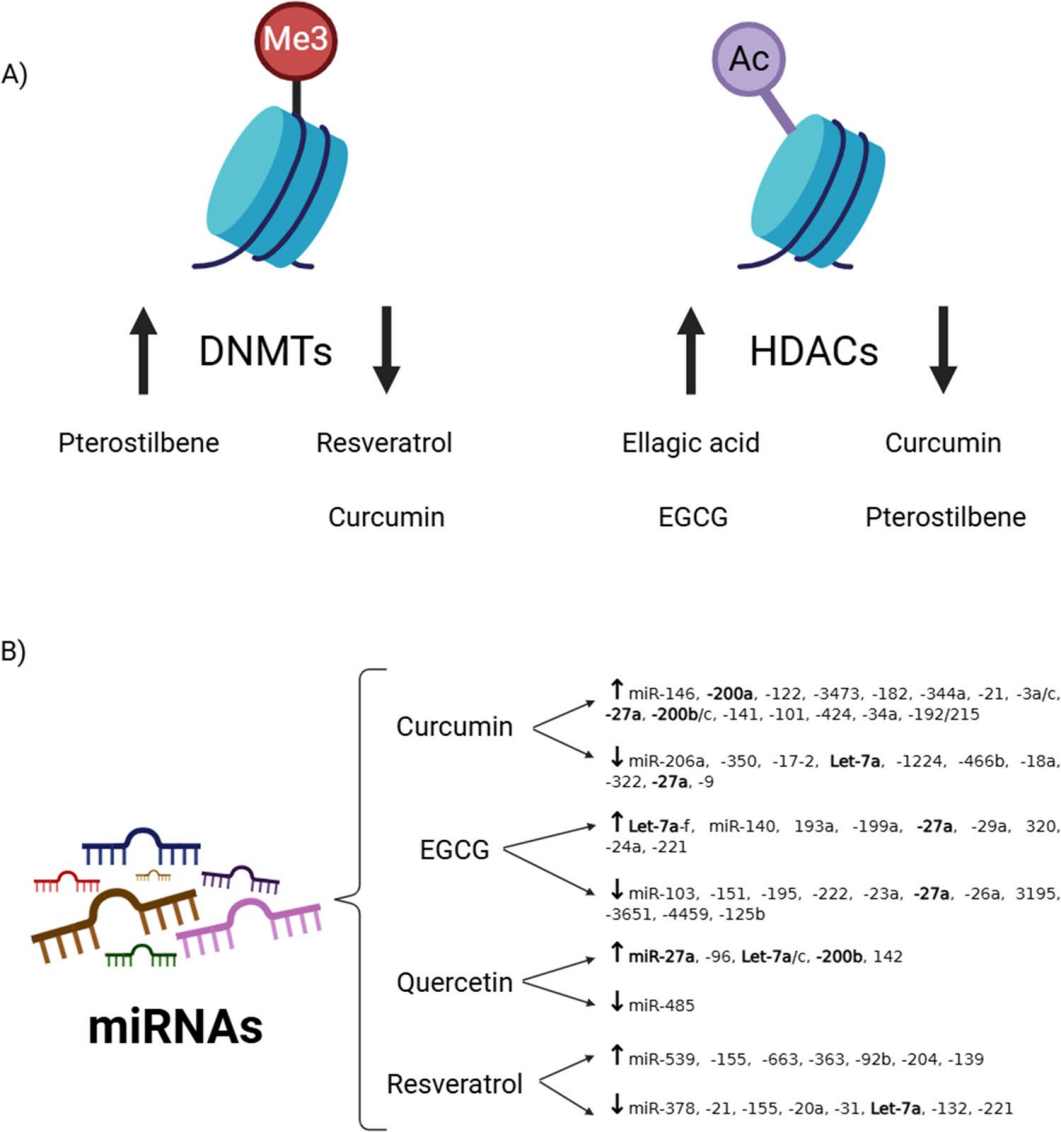
Graphical abstract summarizing the most relevant effects of single polyphenol molecules on epigenetic mechanisms: DNA methylation, histone acetylation, and miRNA expression. **A** Specific changes of single polyphenolic compounds at DNA methylation and histone acetylation indicating an up-regulation (arrow up) or a down-regulation (arrow down) of DNMTs and HDACs expression. **B** Changes in miRNA expression pattern of most relevant single polyphenolic compounds. Reported evidence includes both in in vitro and in vivo studies. DNMT, DNA methyltransferases; EGCG, epigalocatequina-3-galato HDACs, histone deacetylases; miRNAs, microRNAs. Image created with BioRender (https://www.biorender.com/)

Figure 1A summarizes the most relevant results induced by single polyphenolic compounds on epigenetic mechanisms of DNA methylation and histone acetylation.

### Phenolic compounds regulate the expression of specific short non-coding RNAs: microRNAs

Chromatin architecture regulates transcriptional dynamics in response to environmental conditions. However, transcriptional activity is not limited to changes in DNA methylation or histone modifications but also includes the synthesis of non-coding RNAs such as microRNAs (miRNAs). MicroRNAs are short non-coding RNAs that regulate gene expression at post-transcriptional level by binding to target mRNAs and repressing their translation (extensively reviewed in [14]). Nutrients and food components, such as phytochemicals, can influence gene expression, at least in part, through miRNA regulation. Providing that miRNAs can be secreted in extracellular vesicles, their physiological impact could also be transferred at a global systemic level [22].

Polyphenols can stimulate tumor suppressor genes and downregulate inflammation-related genes by specifically altering miRNA expression pattern. This regulation has been extensively studied and reported in available evidences (reviewed in [40, 90]). Indeed, these studies suggest that the well-known anti-inflammatory and antioxidant effects of polyphenols seem to be mediated through specific miRNA regulation. Most reported studies have been performed in vitro models (specifically in cell cultures) although other in vivo and clinical studies have also investigated through a more translational perspective whether these anti-inflammatory/antioxidant effects are related to the specific polyphenol-dependent regulation of miRNAs [86]. Indeed, anti-tumoral, anti-proliferative and anti-inflammatory effects of polyphenols frequently observed in cancer cellular models, probably arise from the effects of polyphenols on the expression of a low number (but critical) of endogenous miRNAs with potential to impact cell transcriptome at a local level. Particularly, in this regard, some of the most representative phenolic compounds specifically affect single miRNA expression (see Table 3). Worth mentioning are let-7a, miR-200a, miR-27a, whose expression levels have been reported to be affected in different models by single polyphenolic compounds quercetin [121], curcumin [44] or EGCG [92]. More specifically, expression of miR-200a has been found to be up-regulated in cancer models (hepatocellular, colorectal, pancreas) by quercetin and curcumin. Similarly, let-7a expression is frequently regulated by polyphenols in a positive manner although differential effects have been observed in several models and depending on the polyphenol type: While curcumin and resveratrol down-regulate let-7a expression in neurological disorders and increase the anti-inflammatory interleukin IL-10 in colitis [5, 101], quercetin and EGCG up-regulate let-7a expression [7, 92].

**Table 3:**
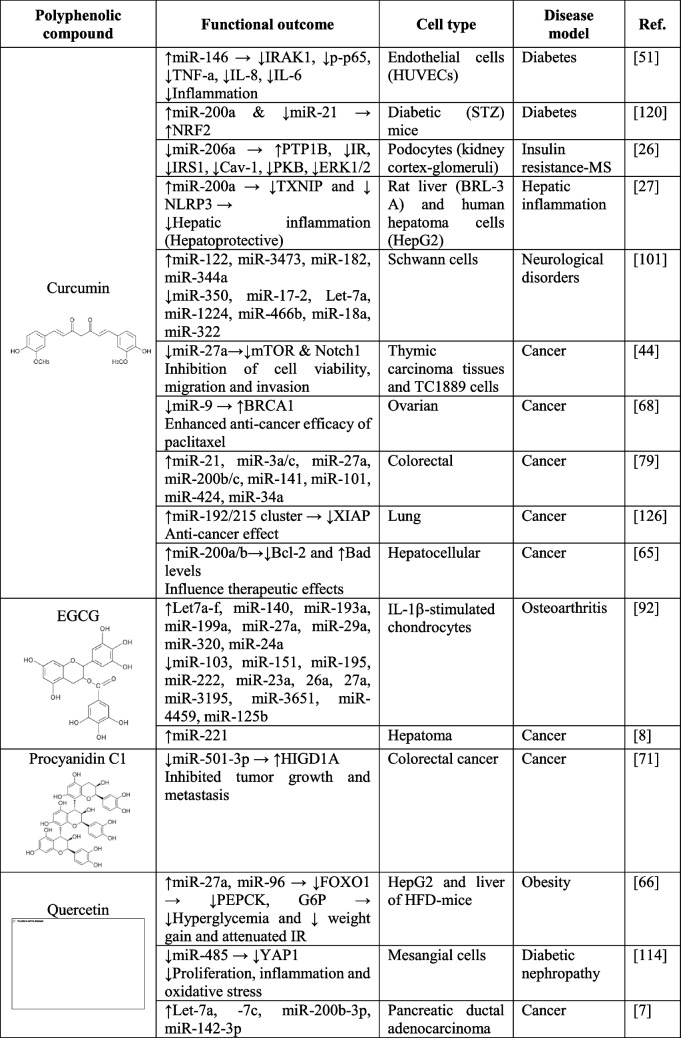

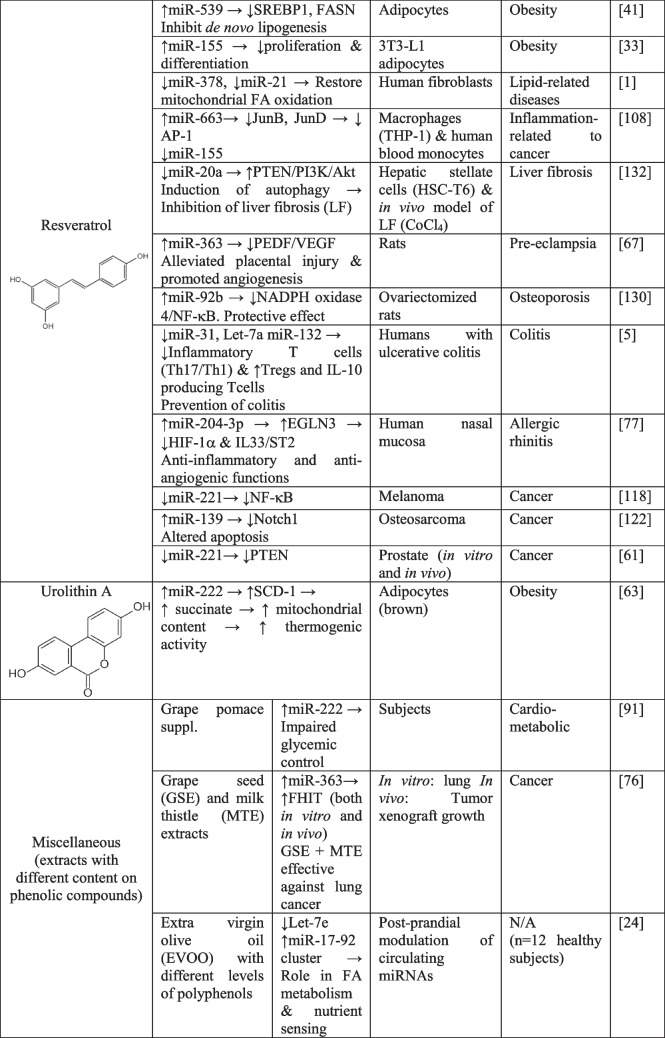
Effects of single compounds of polyphenols origin on miRNA expression and their regulatory actions in several models of human diseases

*EGCG*, epigallocatechin-3-gallate; *IR*, insulin resistance; *STZ*, Streptozotocin; *N/A*, non-applicable

Metabolic inflammation associated with obesity is mainly triggered by altered insulin signaling (insulin resistance). How dietary components, among polyphenols, influence miRNA expression linked to insulin signaling control is a matter of study in the context of precision nutrition (reviewed in [18]). For instance, quercetin increases miR-27a expression, which is responsible for the down-regulation of Forkhead Box 1 (*Foxo1)*, Phosphoenolpyruvate Carboxykinase (*Pepck*) and Glucose-6-Phosphate Dehydrogenase (*G6ph)*, which leads to a decrease in hyperglycemia, body weight gain and improvements in insulin resistance in human hepatocytes and liver of high-fat diet-fed mice [66]. Curcumin also decreased miR-27a expression in two cellular cancer models (thymic and colorectal) which led to inhibition of cell viability and invasion capacity [44, 79]. Further information concerning the analyzed studies in this review are detailed in Table 3. Metabolites of phenolic compounds can also induce changes in miRNA expression: particularly urolithin A, a metabolite resulting from ellagitannins transformation, has been found to induce miR-124 producing brown adipocyte differentiation [63].

Most of these reported studies have specifically identified a set of miRNAs whose expression is mostly up-regulated after polyphenols treatment (in vitro studies) or intake (in vivo and clinical studies). The most relevant findings regarding the effects of specific polyphenols in cancer, metabolic diseases and inflammation-related disorders are summarized in Table 3. Using bioinformatic tools, candidate miRNA-target genes (mRNAs) were identified, and subsequent validation experiments either confirmed or discarded specific miRNA-mRNA regulatory axes for particular disease phenotypes, mainly: cancer, inflammation and metabolic conditions. However, addressing a direct “cause-effect” of polyphenols on miRNAs expression has some caveats such as the lack of reproducibility between studies (inter-individual and experimental variability) and differences in the polyphenolic compounds and concentrations used. Figure 1B illustrates the miRNA expression pattern affected upon polyphenolic single molecules treatment.

Notably, studies involving nutritional interventions provide direct evidence of polyphenol-induced regulatory effects in humans [100]. For instance, Tomé-Carneiro et al*.*, [109] found that resveratrol intake (8.1 mg) altered miRNA expression patterns in peripheral blood mononuclear cells (PBMCs) in men with metabolic syndrome features. Interestingly, resveratrol intake upregulated several miRNAs (miR-21, miR-181b, miR-663, miR-30c) that inhibited inflammatory markers interleukin-6 (IL-6), tumor necrosis factor-alpha (TNF-α) and interleukin 1-beta (IL-1β). On the contrary, resveratrol downregulated miR-155, which activated toll-like receptors (TLR), a family of pattern recognition receptors that play a crucial role in the innate immune system, and NF-$\upkappa$B signaling. Globally, the resveratrol intake contributed to these cell-specific effects promoting the decrease of systemic inflammation and the improvement of MS features. Polyphenols induce specific alterations on single targets [i.e., cyclin dependent kinase 4 (CKD4), vascular endothelial growth factor A (VEGFA), hypoxia inducible factor 1 subunit alpha (HIF-1α), SIRT] and pathways (phosphatase and tensin homolog, PTEN/Akt) that are important in diabetic complications, through targeting miRNAs. However, their efficacy should be addressed in detail in future clinical studies [74]. Other studies addressing the positive effects of resveratrol on cancer and inflammation are reviewed in [107].

Interestingly, some of the described effects of polyphenols on DNA methylases and acetylases (which carry out DNA methylation and histone acetylation, respectively) could be mediated through the regulatory action of miRNAs. Indeed, miRNAs can revert aberrant methylation in cancer by directly targeting DNA and histone methyl-transferases [34, 112] and acetylases [83]. Specific miRNA expression is also dependent on microbial community, which is responsible for the synthesis of polyphenol-derived metabolites also with biological activities [16]. In overall, these findings suggest that functional effects of polyphenols are reliant and finely tuned by miRNAs expression.

### Limitations

Despite well-reported positive effects of polyphenols in overall physiological balance and human health, some limitations arise. An important caveat on translational interpretations to humans concerns the experimental conditions of the in vitro and in vivo studies (doses, treatment length) which might not be relevant regarding the bioavailability knowledge on polyphenol metabolism in humans. Positive effects of polyphenols in (clinical) humans seem to accumulate over time as long as their dietary intake is within standard dietary levels obtained from regular foods consumption. However, it is difficult to simulate this accumulative effect over time in basic (in vitro and in vivo) studies due to technical and experimental differences (concentrations/doses of single molecules or enriched extracts, bioavailability, species). A feasible approach to overcome these limitations could rely on applying an acute/single treatment with a (supra)physiological concentration(s) of polyphenolic molecules (in vitro) or the administration of a relatively high (non-toxic) dose(s) of polyphenolic compounds for a limited period of time. Nevertheless, existing differences prevent a full translation to humans of the results obtained in these preclinical approaches, which warrant an optimal experimental design trying to represent the most reliable human setting.

### Future directions

Further understanding of epigenetic mechanisms of phenolic compounds represent a current challenge in order to develop nutraceuticals, food supplements and new food products with well-supported molecular mechanisms endorsing their use in human health. The epigenetic reprogramming resulting from the treatment or administration of polyphenolic compounds can be monitored in controlled settings such as cell lines or animal models. On the other hand, in vivo studies (i.e., intervention studies in humans) can provide insights into how dietary polyphenols affect epigenetic reprogramming in a more complex biological context, including the effects on metabolism and interactions with other dietary components. In fact, several high-throughput technologies are available to characterize the changes induced by these treatments. For instance, methylated DNA immunoprecipitation coupled to sequencing (MeDIP-seq) is used to study 5-methylcytosine (5mC) or 5-hydroxymethylcytosine (5hmC) modifications in DNA. Chromatin immunoprecipitation coupled to sequencing (ChIP-seq) and assay for transposase- accessible chromatin coupled to sequencing (ATAC-seq) are commonly employed to analyze histone modification and chromatin accessibility. Additionally, RNA sequencing (RNA-seq) can be used to profile miRNAs. Together, these technologies provide valuable insight regarding the precise effects of polyphenols on epigenetic reprogramming, which is essential for understanding the beneficial effects of these compounds. Recently, circulating free DNA (cfDNA) and nucleosomes obtained from liquid biopsies have been utilized for epigenetic profiling in cancer subtyping, representing a significant advancement in the field [11, 12]. Indeed, all these could represent novel advances and future avenues for addressing specific effects of polyphenols in epigenetics.

## Conclusion

Polyphenols constitute an important tool to investigate epigenetic reprogramming, including alterations in DNA methylation, histone modifications and miRNA expression. For example, curcumin modifies histone acetylation status, decreases DNMT and miR-27a expression, thus conferring health benefits against cancer. Resveratrol, EGCG and pterostilbene inhibit DNA methyltransferases, histone deacetylases and specifically regulate miRNA expression (i.e., let-7a, miR-27a, miR-221). Given their bioactive potential, phenolic compounds might be considered as dietary micronutrients or ingredients for the development of new food products or nutraceuticals as a complement to a healthy-balanced diet in cancer, inflammation and metabolic disorders. Standardizing dosages and formulations is also essential to enhance bioavailability and ensure effective translation into clinical applications. Additionally, longitudinal studies are needed to determine whether polyphenol-induced epigenetic changes are transient or sustained over time, which will be crucial for assessing their long-term impact on human health. However, polyphenols specifically modulate epigenetic mechanisms at a cellular level and future studies should focus on establishing the precise molecular pathways through which polyphenols exert epigenetic control to clarify their mechanisms of action. Given their potential to regulate human health through epigenetic mechanisms, phenolic compounds represent promising candidates for personalized nutrition and therapeutic strategies. However, rigorous clinical validation is essential before their widespread adoption in epigenetic-based interventions. Additionally, phenolic compounds and their metabolites could serve as biomarkers of food intake and as bioactive components in next-generation nutraceutical products.

## Data Availability

No datasets were generated or analysed during the current study.
